# Altered Synaptic and Extrasynaptic NMDA Receptor Properties in Substantia Nigra Dopaminergic Neurons From Mice Lacking the GluN2D Subunit

**DOI:** 10.3389/fncel.2018.00354

**Published:** 2018-10-11

**Authors:** Paul G. Morris, Masayoshi Mishina, Susan Jones

**Affiliations:** ^1^Department of Physiology, Development and Neuroscience, University of Cambridge, Cambridge, United Kingdom; ^2^Brain Science Laboratory, The Research Organization of Science and Technology, Ritsumeikan University, Shiga, Japan

**Keywords:** NMDA receptor, GluN2D subunit, GluN2B subunit, substantia nigra, dopamine neuron, NMDAR-EPSC, tonic NMDAR current

## Abstract

*N*-methyl-D-aspartate receptors (NMDARs) are ubiquitously expressed in the mammalian brain and are essential for neuronal development, survival and plasticity. GluN2 subunit composition has a profound effect on the properties of NMDARs. In substantia nigra dopaminergic (SNc-DA) neurons, pharmacological experiments suggest that the relatively rare GluN2D subunits form functional synaptic and extrasynaptic NMDARs. Given the importance of establishing this point, mice lacking the GluN2D subunit (*Grin2D*-null) were used in this study to further explore the contribution of the GluN2D subunit to NMDAR responses. Significantly less DQP-1105-sensitive NMDAR-EPSC and significantly more ifenprodil-sensitive NMDAR-EPSC was observed in SNc-DA neurons from *Grin2D*-null mice, indicating that in these animals a small population of synaptic GluN2D subunits is replaced with GluN2B. Significantly larger currents were seen in response to higher concentrations (1–10 mM) of NMDA in SNc-DA neurons from *Grin2D*-null mice, as well as significantly more desensitization: these data are consistent with the presence of GluN2D-containing whole-cell NMDARs in SNc-DA neurons, with low conductance and little desensitization. Brief applications of NMDA evoked responses that were significantly less sensitive to DQP-1105 in slices from *Grin2D*-null mice. Tonic NMDAR activity in response to ambient extracellular glutamate, determined by the sensitivity of tonic current to D-AP5 (50 μM), was significantly less in SNc-DA neurons from *Grin2D*-null mice. In the presence of the glutamate transporter blocker TBOA (30 μM), the D-AP5-sensitive current was also significantly less in *Grin2D-*null mice. Taken together, these data support the evidence for GluN2D subunit expression in functional NMDARs at both synaptic and extrasynaptic locations in SNc-DA neurons.

## Introduction

*N*-methyl-D-aspartate receptors (NMDARs) are ubiquitous at mammalian brain excitatory synapses, where they are opened by co-incident detection of glutamate, glycine/D-serine and depolarization to relieve their Mg^2+^ ion block (Traynelis et al., 2010). The functional and pharmacological properties of NMDA receptors are determined by their subunit composition (Stern et al., 1992; Wyllie et al., 1996, 2013; Paoletti et al., 2013). GluN2 subunits are the main determinants of NMDA receptor functional diversity, and the four GluN2 subunits, GluN2A - GluN2D, each confer different properties.

The distribution of NMDAR GluN2 subunits throughout the brain alters during postnatal development (Monyer et al., 1994). GluN2B expression is high at birth, particularly in the cortex and hippocampus, and gradually declines to a lower level in adulthood. Conversely, GluN2A expression is low at birth and increases throughout development such that both GluN2A and GluN2B-containing NMDARs are expressed in mature cortical neurons (Monyer et al., 1994; Wenzel et al., 1995, 1997). GluN2C is expressed later in development, and is mostly confined to the cerebellum and olfactory bulb (Watanabe et al., 1992; Monyer et al., 1994; Wenzel et al., 1997). GluN2D subunit expression is prominent in the brainstem and diencephalon in neonates but decreases throughout development; in mature neurons, it is reported in some cerebellar nuclei, the striatum, the olfactory bulb, substantia nigra dopaminergic (SNc-DA) neurons, subthalamic nucleus (STN) neurons, and hippocampal interneurons (Watanabe et al., 1993, 1994; Monyer et al., 1994; Standaert et al., 1994; Wenzel et al., 1995, 1997; Misra et al., 2000; Brickley et al., 2003; Lozovaya et al., 2004; Jones and Gibb, 2005; Brothwell et al., 2008; Harney et al., 2008; Suárez et al., 2010; Mullasseril et al., 2010; Swanger et al., 2015; von Engelhardt et al., 2015; Perszyk et al., 2016).

All of these studies have at least in part relied on pharmacological tools. Ifenprodil has a 140-fold preference for GluN2B over GluN2A (Gallagher et al., 1996), and has been used extensively to study native GluN2B subunits in brain neurons. Ifenprodil inhibition is incomplete and non-competitive (Williams, 1993; Mott et al., 1998; Amico-Ruvio et al., 2011; Tajima et al., 2016). DQP-1105 was developed more recently (Mosley et al., 2010; Ogden and Traynelis, 2011) and is a non-competitive antagonist that binds to the S2 region of the GluN2D subunit, blocking a conformational change necessary for channel opening; DQP-1105 is around 50 times more selective for GluN2C/D-containing NMDARs over those containing GluN2A or B (Acker et al., 2011). None-the-less, the pharmacological tools alone for identifying the subunit composition of native NMDARs are limited.

Based on their physiological and pharmacological properties, NMDARs in SNc-DA neurons at around P7 are thought to be arranged in two populations of diheteromeric receptor: those composed of GluN1/GluN2B and those composed of GluN1/GluN2D. By P21, SNc-DA neurons are thought to express NMDARs in a triheteromeric GluN1/GluN2B/GluN2D configuration (Jones and Gibb, 2005; Brothwell et al., 2008; Suárez et al., 2010). The limited selectivity of pharmacological tools make unequivocal conclusions based on ifenprodil and UBP141 (Brothwell et al., 2008; Suárez et al., 2010) difficult. In this study, mice lacking the GluN2D subunit (*Grin2D*-null mice) have been used to bring a new tool to determining the contribution of GluN2D to synaptic and extrasynaptic NMDAR responses. Specifically, we aimed to compare the pharmacological properties of synaptic NMDARs, defined by their sensitivity to neurotransmitter released in response to a single action potential, and extrasynaptic receptors, that are activated in response to exogenous NMDA, when glutamate uptake is compromised, or in response to glutamate release from glia (Sah et al., 1989; Asztely et al., 1997; Angulo et al., 2004; Fellin et al., 2004; Le Meur et al., 2007). Our data support the idea that GluN2D subunits contribute to both synaptic and extrasynaptic NMDARs in SNc-DA neurons throughout postnatal development.

## Materials and Methods

### Animals

**FIGURE 1:**
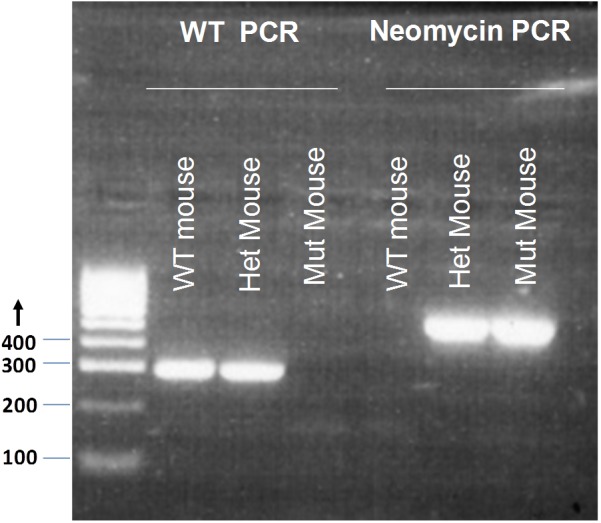
Genotyping of *Grin2D*-wild type and *Grin2D*-null mice. An example electrophoresis gel, showing PCR products from genotyping of homozygous *Grin2D*-wild type (WT), heterozygous (Het), and homozygous *Grin2D*-null (Mut) mice, alongside HyperLadderTM 100bp molecular weight markers from Bioline (London, United Kingdom).

C57BL/6 mice were obtained from Charles River UK. *Grin2D*-null and *Grin2D*-wild type (WT) littermate mice were bred in house from *Grin2D*-heterozygous mice obtained from the Riken BRC, through the National Bio-Resource Project of the MEXT, Japan, and crossed with each other or with C57 mice to generate both *Grin2D*-null and *Grin2D*-WT mice on the same background. The mutant line was originally generated by disruption of the exon encoding the GluN2D transmembrane M4 region by insertion of a neomycin phosphotransferase gene (Ikeda et al., 1995). These mice grow and mate normally and exhibit no major structural brain abnormalities (Ikeda et al., 1995; Miyamoto et al., 2002). All experiments were performed using these animals at P5-23, in accordance with The Animals (Scientific Procedures) Act 1986 and local Animal Welfare and Ethical Review Board approval. All animals were provided with ad libitum access to food and water, and kept on a 12 h alternating light/dark cycle. The mice were genotyped from ear biopsies or crude tail tissue sample lysates using the following primer set:

GluR4 WT F 5′-GTGCTCCTAATAAGTGACTCTGA-3′ and GluR4 WT R 5′-CCTCCTCGCTCCCTTTCTT-3′ produce a 289 bp product for the WT locus. NeoF 5′-CGGTGCCCTGAATGAACT-3′ and NeoR 5′-CACGGGTAGCCAACGCTATG-3′ produce a 618 bp product for the mutant (Mut) locus (**Figure 1**). Tissue samples of breeding pairs and selected offspring were outsourced for genotyping to the Andrew Huxley Genotyping Facility, University College London. A total of 186 mice were used for the experiments in this study; 98 *Grin2D*-null mice and 88 WT mice (including 43 *Grin2D*-WT mice and 45 C57Bl/6 mice.

### Brain Slice Preparation

Mice were decapitated under isoflurane anesthesia and the brain was removed into ice cold slicing solution composed of (mM): NaCl 75; sucrose 100; glucose 25; NaHCO_3_ 25; KCl 2.5; CaCl_2_ 1; MgCl_2_ 4; NaH_2_PO_4_ 1.25; kynurenic acid 0.25, maintained at pH 7.4 by bubbling with 95% O_2_ and 5% CO_2_. Horizontal midbrain slices (230–300 μm) containing the substantia nigra were prepared using a Campden 7000smz Vibrating Microtome (Campden Instruments, United Kingdom). Slices were transferred to a submersion incubation chamber containing a modified recording solution of composition (mM): NaCl 125; glucose 25; KCl 2.5; NaHCO_3_ 26; NaH_2_PO_4_ 1.26, MgCl_2_ 4; CaCl_2_ 1, bubbled with 95% O_2_ and 5% CO_2_ and maintained at 30°C for 1–6 h prior to use.

### Electrophysiology

Brain slices were transferred to the stage of an Olympus BX51W upright microscope and SNc dopamine neurons were viewed at a magnification of x600 using differential interference contrast optics. The chamber was perfused at 2–3 ml/min with recording solution at 30 ± 2°C (as above but with 10 mM glucose and 1 mM MgCl_2_ or, for recordings at -50 mV, 0.1 mM MgCl_2_; pH 7.4 with 95% O_2_/5% CO_2_). Patch pipettes were pulled from thin walled borosilicate glass (GC150F, Harvard apparatus, Kent, United Kingdom) to a resistance of 1–3 MΩ and filled with pipette solution containing (mM): CsCH_3_SO_3_ 130; CsCl 5; NaCl 2.8; HEPES 20; EGTA 5; CaCl_2_ 0.5; MgCl_2_ 3; Mg-ATP 2; Na-GTP 0.3; pH ∼7.2. Cells were voltage-clamped to -50 mV (in 0.1 mM MgCl_2_) or +40 mV (in 1 mM MgCl_2_) using an Axopatch 200B patch-clamp amplifier or a HEKA EPC9 amplifier. For current-voltage experiments, 1.3 mM MgCl_2_ was used and the membrane potential was changed to steady-state values between +40 mV and -80 mV. Membrane current was low pass filtered at 2 kHz and sampled at 20 kHz using a Micro 1401 controlled by Spike 2 (Version 4) software (Cambridge Electronic Design, Cambridge, United Kingdom). Series resistance (typically 3–6 MΩ) was compensated by up to 40%. SNc dopamine neurones were identified by their anatomical location and the presence of a prominent inward current (*I*_sag_) during a voltage step from -60 to -110 mV. This current is representative of a hyperpolarization-activated inward (*I*_h_) current (Washio et al., 1999; Neuhoff et al., 2002; Margolis et al., 2006).

Synaptic currents were evoked using a bipolar stainless steel electrode (Frederick Haer and Co., United States) placed within the SN rostral to the recorded cell at an approximate distance of 0.5 mm, and stimuli (200 μs duration; stimulation intensity, 50–140 μA) were applied at 0.1 Hz in the presence of DNQX (10–20 μM), picrotoxin (50 μM) and glycine (10 μM) (all from Sigma–Aldrich, United Kingdom). For pharmacological experiments, peak EPSC amplitude was determined from the average of at least 100 s of data (10 EPSCs) prior to drug application and at least 100 s of data following perfusion of antagonist for a minimum of 10 min. For current-voltage relationships, peak EPSC amplitude was determined from the average of at least 50 s of data (5 EPSCs) at different steady state membrane potentials; the ratio of current at -60 mV to current at +40 mV within the first 15 min of each experiment was determined as a measure of the degree of Mg^2+^ block at hyperpolarized potentials.

For measurement of the time constant of NMDAR-EPSC decay, 10–20 EPSCs were averaged and the decay from the peak of the response to the plateau was fit with a two component exponential decay function (in GraphPad Prism):

$$I(t)=A_{1}e^{-\frac{t}{\tau_{1}}}+A_{2}e^{-\frac{t}{\tau_{2}}}$$

The weighted time constant (τ_W_) was calculated as:

$$\tau_{\text{w}}={\text{τ}}_{1}(\frac{A_{1}}{A_{1}+A_{2}})+{\text{τ}}_{2}(\frac{A_{1}}{A_{1}+A_{2}})$$

Whole-cell NMDAR-mediated currents were evoked in the presence of glycine (10 μM), picrotoxin (50 μM), and tetrodotoxin (100 nM), either by application of NMDA (10 μM–10 mM) to the bath perfusion or by application from a picospritzer (0.5 mM for 20 s, 10–15 psi, every 200 s). Peak amplitude (and in some experiments percent desensitization; the decline in current from peak to plateau during the continued presence of NMDA) was measured. The pharmacology of whole-cell NMDAR-mediated currents was determined by comparing the average of three control NMDAR peak current amplitudes with the peak current amplitude of two responses to NMDA after a 10 min perfusion with DQP-1105.

### Statistics

Data are expressed as mean ± standard error (SE); the ‘n’ values refer to the number of cells (which also corresponds to the number of slices). To test whether data sets showed a normal distribution, the Shapiro–Wilk normality test was used; data sets that were not significantly different from a normal distribution were analyzed using the Student’s one- or two-tailed *t*-test; non-parametric tests (as reported in the text) were used for two groups that were not both normally distributed. For three or more groups of data, one-way ANOVA with Tukey post-tests (or non-parametric tests) was used. For statistical comparisons the significance level was set to 0.05. Statistical analysis was performed using Prism 4 (version 4.01; Prism 7, version 7.04 was used to fit the concentration-response curve; GraphPad Software (La Jolla, CA, United States).

### Materials

DNQX, D-AP5, glycine, NMDA, ifenprodil and picrotoxin were purchased from Sigma–Aldrich, United Kingdom. The glutamate transporter inhibitor DL-threo-benzyloxyaspartic acid (TBOA), DQP-1105 and tetrodotoxin (TTX) were purchased from Tocris, United Kingdom.

## Results

### Synaptic NMDAR Responses in *Grin2D*-Null Mice

**FIGURE 2:**
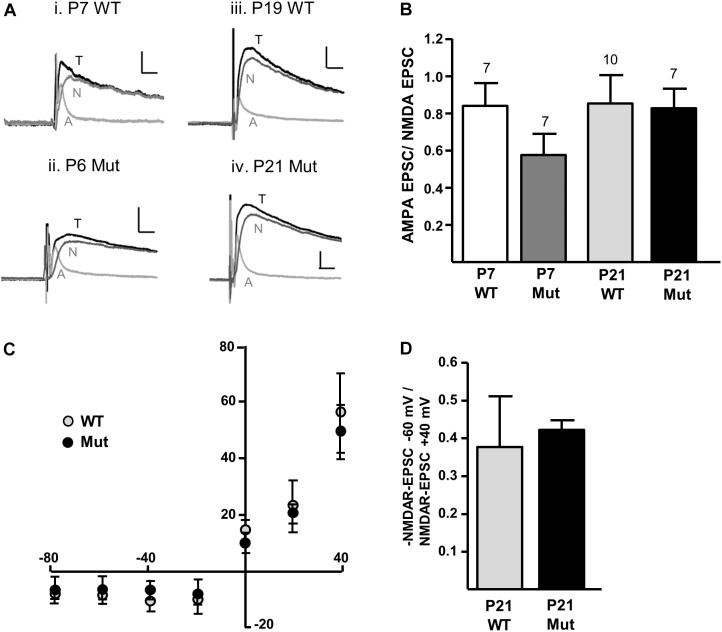
Constitutive expression of NMDARs in *Grin2D*-null mice. **(A)** Example recordings from SNc-DA neurons in slices from (i) *Grin2D*-WT mouse aged P7 (ii) *Grin2D*-null mouse aged P6 (iii) *Grin2D*-WT mouse aged P19 and (iv) *Grin2D*-null mouse aged P21. Scale bar is 40 pA and 10 ms for all recordings. T, total EPSC; A, AMPAR-EPSC; N, NMDAR-EPSC. **(B)** Bar graph shows the quantified ratio of AMPAR-EPSC to NMDAR-EPSC at P7 and P21 in WT and Grin2D-null mice (numbers of cells/ slices indicated; from five *Grin2D*-WT mice; six C57 mice and ten *Grin2D*-null mice; *P* = 0.27, Kruskal–Wallis test). **(C)** Graph shows the amplitude of NMDAR-EPSCs (pA) plotted against steady state membrane potential (mV) in recordings from P21 WT or *Grin2D*-null mice (six C57 mice and seven *Grin2D*-null mice). **(D)** Bar graph shows the quantified ratio of NMDAR-EPSC current at –60 mV to +40 mV in seven C57 mice and five *Grin2D*-null mice (*P* = 1.0, Mann–Whitney test).

We first investigated whether removal of the *Grin2D* gene caused any change in the overall expression of NMDARs at the synapse, relative to AMPARs (A/N ratio). To determine the A/N ratio, picrotoxin (50 μM) and glycine (10 μM) were added to the perfusion, cells were voltage-clamped to +40 mV and EPSCs of a stable amplitude were recorded for at least 300 s before addition of D-AP5 (50 μM) to the perfusion. At least 10 EPSCs immediately before (total EPSC), and 10 EPSCs in the presence of D-AP5 (AMPAR-EPSC) were averaged and the AMPAR-EPSC was subtracted from the total-EPSC to give the NMDAR-EPSC (**Figure 2A**). Peak amplitudes of each component were measured 8–15 ms following the stimulus artifact. In mice aged P5–P8 (‘P7’), A/N values were 0.840 ± 0.124 in *Grin2D*-WT (*n* = 7), and 0.577 ± 0.113 in *Grin2D*-null mice (*n* = 7). In mice aged P17–P23 (‘P21’), A/N values were 0.721 ± 0.079 in WT mice (*n* = 10), and 0.827 ± 0.104 in *Grin2D*-null mice (*n* = 7). There was no significant difference in A/N EPSC ratios (*P* = 0.27, Kruskal–Wallis test; **Figure 2B**). We also examined the current-voltage relationship of NMDAR-EPSCs in the presence of 1.3 mM MgCl_2_, to determine whether the Mg^2+^ sensitive block was altered in *Grin2D*-null mice. Current voltage relationships are shown in **Figure 2C** for SNc-DA neurons from P21 WT and *Grin2D*-null mice. The NMDAR-EPSC amplitude at -60 mV versus +40 mV is shown in **Figure 2D**; the ratio shows greater variability in the WT group (0.38 ± 0.13, *n* = 7) compared with the *Grin2D*-null group (0.42 ± 0.03, *n* = 5), but there was no significant difference between the mean ratios (*P* = 1.0, Mann–Whitney test).

**FIGURE 3:**
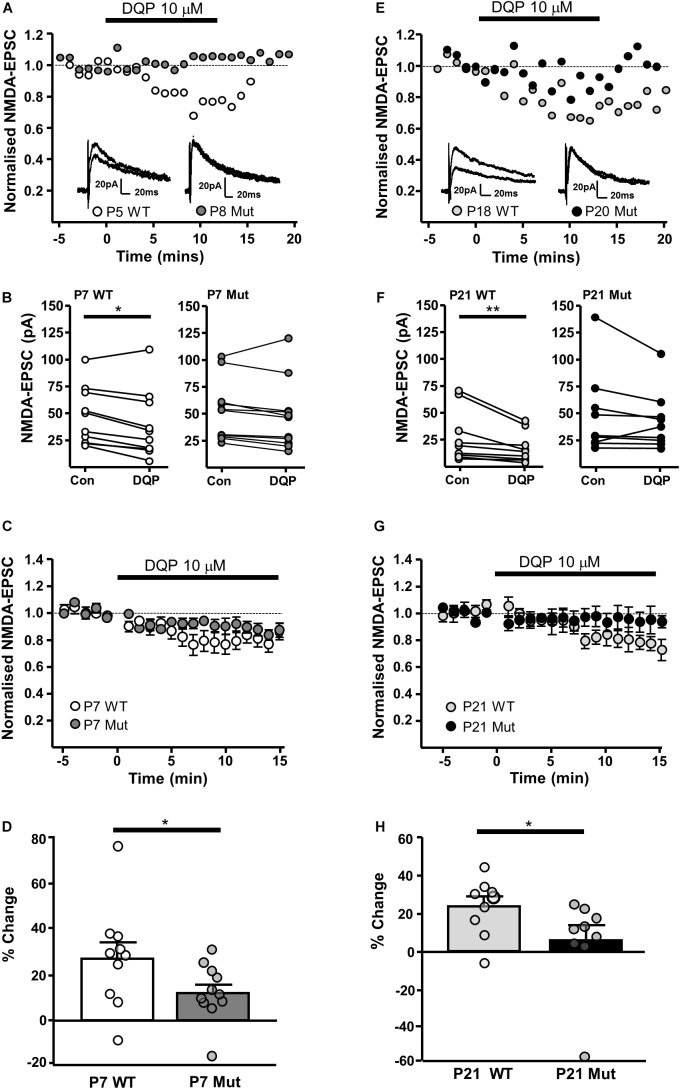
Decreased sensitivity of synaptic NMDARs to DQP-1105 in *Grin2D*-null mice. **(A)** Example recordings from SNc-DA neurons in slices from a *Grin2D*-WT mouse aged P5 and a *Grin2D*-null (Mut) mouse aged P8. **(B)** Graph of NMDAR-EPSC (pA) before and during perfusion with DQP-1105 in P7 WT and *Grin2D*-null mice (^∗^*P* = 0.027, Wilcoxon Signed Rank test). **(C)** Combined data from SNc-DA neurons in slices from WT and *Grin2D*-null mice aged P7. **(D)** Bar graph shows % inhibition with DQP-1105 in WT and *Grin2D*-null mice at P7 (data points from individual cells are superimposed; ^∗^*P* = 0.024, one tailed Mann–Whitney test). **(E)** Example recordings from SNc-DA neurons in slices from a *Grin2D*-WT mouse aged P18 and a *Grin2D*-null mouse aged P20. **(F–H)** The same quantification as in **(B–D)** but from mice aged P21 (in **F**, ^∗∗^*P* = 0.004, Wilcoxon Signed Rank test; in **H**, ^∗^*P* = 0.005, one tailed Mann–Whitney test). Data in **(D,H)** are from seven *Grin2D*-WT mice, nine C57 mice and nineteen *Grin2D*-null mice.

We have previously shown that NMDAR-EPSCs in rat SNc-DA neurons are inhibited by the GluN2D-preferring antagonist UBP141 (Feng et al., 2004; Morley et al., 2005; Monaghan et al., 2012) up to the third postnatal week, consistent with the expression of GluN2D-containing synaptic NMDARs, potentially in triheteromeric arrangements with GluN1 and GluN2B (Brothwell et al., 2008). We therefore investigated whether the pharmacological properties of synaptic NMDARs would be altered in *Grin2D*-null mice by the GluN2D-preferring antagonist DQP-1105 (10 μM). Example recordings are shown in **Figure 3A** (P7) and **Figure 3E** (P21). We compared the NMDAR-EPSC current amplitude during the baseline recording with the amplitude in the presence of DQP-1105 (**Figures 3B,F**). At P7, there was a significance decrease in current amplitude in WT mice (from 47.2 ± 8.5 pA to 38.6 ± 9.9 pA, *n* = 10; *P* = 0.027, Wilcoxon Signed rank test), but no significant change in *Grin2D*-null mice (51.3 ± 8.3 pA versus 47.1 ± 9.5 pA, *n* = 11; *P* = 0.054, Wilcoxon Signed rank test). At P21, there was also a significance decrease in current amplitude in WT mice (from 30 ± 7.9 pA to 19.8 ± 4.5 pA, *n* = 9; *P* = 0.004, Wilcoxon Signed rank test), but no significant change in *Grin2D*-null P21 mice (48.6 ± 12.8 pA versus 42.9 ± 9.0 pA, *n* = 9; *P* = 0.098, Wilcoxon Signed rank test). The time course of the effect of DQP-1105 is shown in **Figures 3C,G** (NMDAR-EPSCs have been normalized to baseline in each experiment to remove the variability due to activation of different numbers of axons in each experiments). DQP-1105 caused a small and variable degree of inhibition of NMDAR-EPSC amplitude (pA) in slices from P7 WT mice (26.8 ± 6.7%, *n* = 10) and in slices from P21 WT mice (29 ± 5.1%, *n* = 9), suggestive of a variable contribution of GluN2D subunits to NMDAR-EPSCs. Significantly less inhibition was seen in slices from *Grin2D*-null mice at both P7 (12.5 ± 3.9%, *n* = 11; *P* = 0.022, one-tailed Mann–Whitney test; **Figure 3D**) and P21 (3.3 ± 8.4%, *n* = 9; *P* = 0.005, one-tailed Mann–Whitney test; **Figure 3H**), although inhibition of NMDAR-EPSCs from *Grin2D*-null mice was also variable.

**FIGURE 4:**
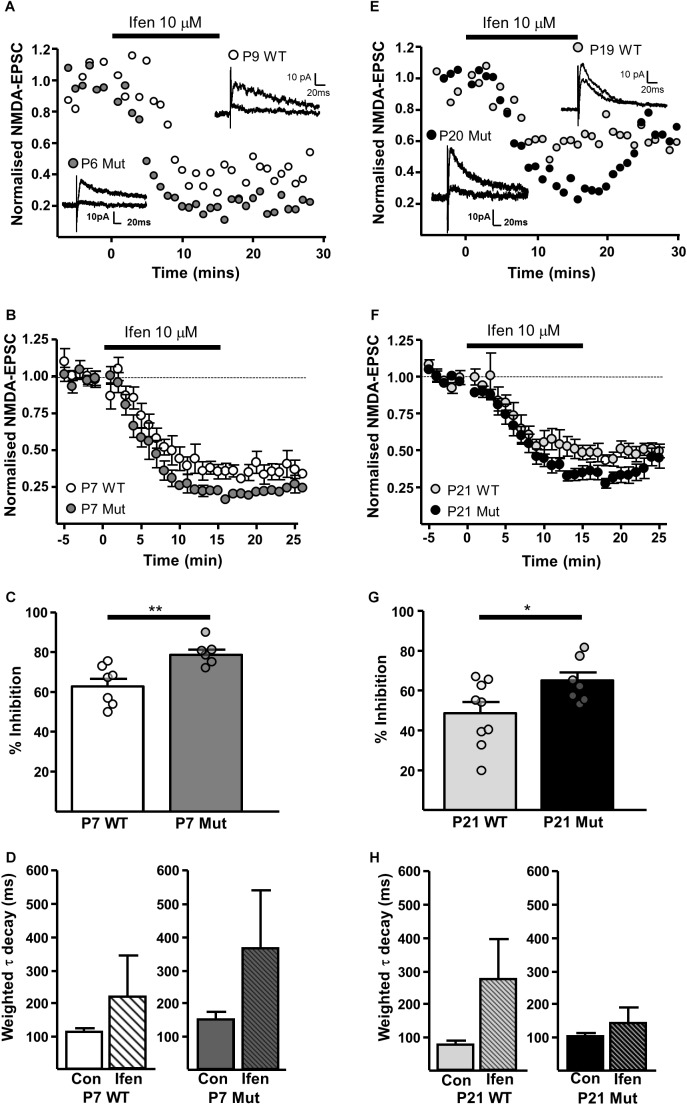
| Increased sensitivity of synaptic NMDARs to ifenprodil in *Grin2D*-null mice. **(A)** Example recordings from SNc-DA neurons in slices from a *Grin2D*-WT mouse aged P9 and a *Grin2D*-null (Mut) mouse aged P6. **(B)** Combined data from SNc-DA neurons in slices from WT and *Grin2D*-null mice aged P7. **(C)** Bar graph shows % inhibition with ifenprodil in WT and *Grin2D*-null mice at P7 (data points from individual cells are superimposed; ^∗∗^*P* = 0.008, Mann–Whitney test). **(D)** Bar graph shows the time constant of the decay of the control NMDA-EPSC and of the NMDA-EPSC component remaining in the presence of 10 μM ifenprodil in SNc-DA neurons from P7 WT (*P* = 1.0, Wilcoxon Signed Rank test) and *Grin2D*-null mice (*P* = 0.3, Wilcoxon Signed Rank test). **(E)** Example recordings from SNc-DA neurons in slices from a WT mouse aged P19 and a *Grin2D*-null mouse aged P20. **(F,G)** The same quantification as in **(B,C)**, but from mice aged P21 (^∗^*P* = 0.04, unpaired *t*-test). **(H)** Bar graph shows the time constant of the decay of the control NMDA-EPSC and of the NMDA-EPSC component remaining in the presence of 10 μM ifenprodil in SNc-DA neurons from P21 WT (*P* = 0.4, Wilcoxon Signed Rank test) and *Grin2D*-null mice (*P* = 0.4, Wilcoxon Signed Rank test). Data in **(B–D,F–H)** are from nine *Grin2D*-WT mice, seven C57 mice and eleven *Grin2D*-null mice.

The GluN2B-preferring antagonist ifenprodil (10 μM), caused significantly more inhibition of NMDAR-EPSCs in SNc-DA neurons from P7 *Grin2D*-null mice (78.8 ± 2.5%, *n* = 6) than in P7 WT mice (62.9 ± 3.7%, *n* = 7; *P* = 0.008, Mann–Whitney test) (**Figures 4A–C**), and in neurons from P21 *Grin2D*-null mice (65.4 ± 4.1%, *n* = 7) than in P21 WT mice (48.8 ± 5.4%, *n* = 9; *P* = 0.04, unpaired *t*-test) (**Figures 4E–G**), suggesting that the deletion of GluN2D subunits results in replacement with GluN2B subunits at synaptic NMDARs in SNc-DA neurons. The data are also consistent with a general developmental decrease from P7 to P21 in diheteromeric GluN1/GluN2B NMDARs in WT SNc-DA neurons (Brothwell et al., 2008). The ifenprodil and DQP-1105 sensitive component of NMDAR-EPSCs in WT and *Grin2D*-null mice is summarized in **Table 1**. The weighted time constant of the decay of control NMDA-EPSCs was not significantly different between *Grin2D*-null mice (150 ± 24 ms, *n* = 5) versus WT (109 ± 10 ms, *n* = 6) at P7 (**Figure 4D**) nor in *Grin2D*-null mice (101 ± 9 ms, *n* = 6) versus WT (75 ± 11, *n* = 8) at P21 (**Figure 4H**). There was no significant difference between the weighted time constant of the decay of control NMDAR-EPSCs versus the NMDAR-EPSC remaining in the presence of ifenprodil, although there was a general trend of an increase in the decay time at both ages suggesting that a slower component remains after ifenprodil block (**Figures 4D,H**).

**Table 1:** Summary of % inhibition of NMDAR-EPSC in SNc-DA neurons from WT and *Grin2D*-null (Mut) mice.

	DQP-1105 (%)	Ifenprodil (%)	Total (rounded)
WT P7	26 ± 8	63 ± 4 *(73)*	89 *(99)*
WT P21	23 ± 5	49 ± 5 *(59)*	72 *(82)*
Mut P7	11 ± 4	79 ± 3 *(89)*	90 *(100)*
Mut P21	5 ± 8	65 ± 4 *(75)*	70 *(80)*


*Mean and SE values have been rounded up or down to the nearest integer. *In parentheses*: % inhibition accounting for a maximum inhibition of 90% with ifenprodil.*

### Extrasynaptic NMDAR Expression in *Grin2D*-Null Mice

**FIGURE 5:**
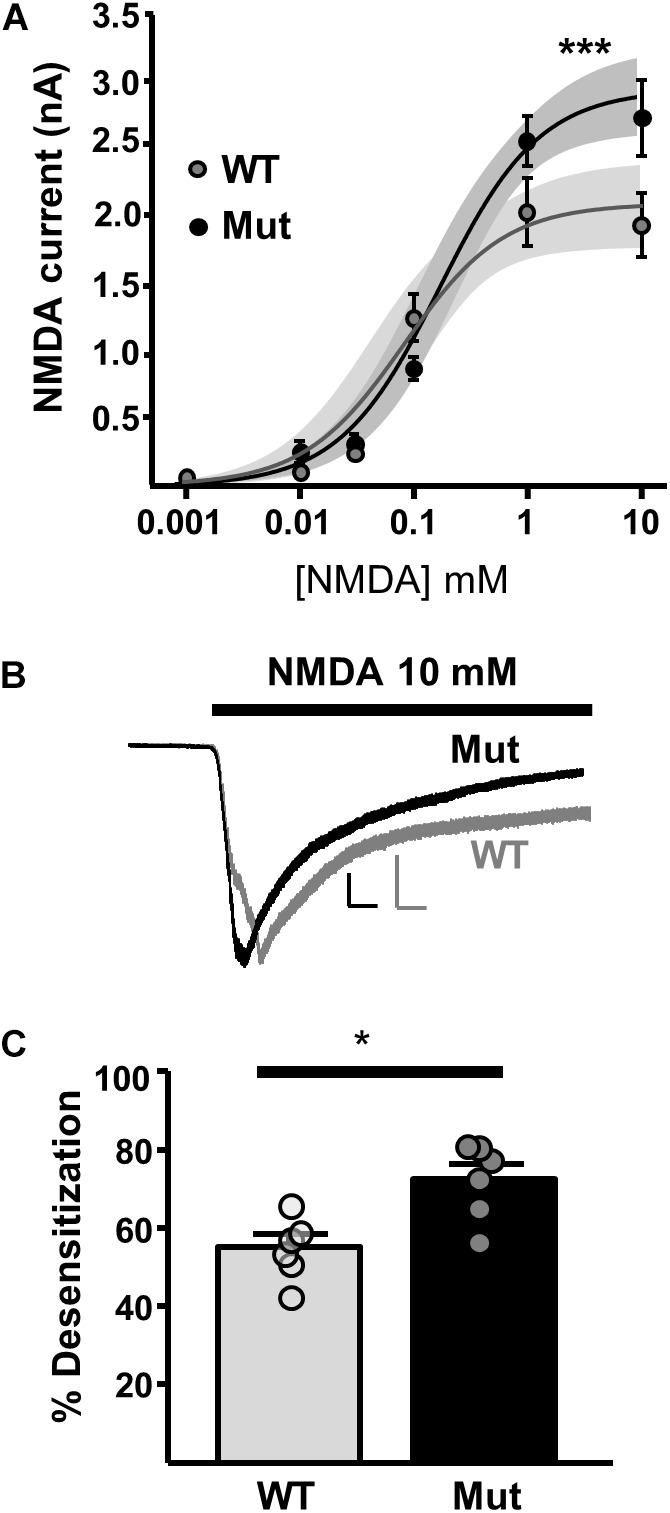
Constitutive expression of whole cell NMDARs in *Grin2D*-null mice. **(A)** Logarithmic concentration-response curves for NMDA-evoked currents in WT (gray; *n* = 24 cells/ slices) and *Grin2D*-null (Mut) mice (black; *n* = 37 cells/slices). A non-linear curve was applied for each genotype using the least squares (ordinary) fit method (solid line). Shaded zones indicate the 95% confidence interval for each curve fit, calculated using the profile likelihood asymmetrical method (Venzon and Moolgavkar, 1988). The curves separated at the highest concentrations of NMDA (^∗∗∗^*P* = 0.0001, extra sum-of-squares *F* test). Data in **(A)** are from 18 *Grin2D*-WT mice, 4 C57 mice and 18 *Grin2D*-null mice. **(B)** Example recordings from SNc-DA neurons in slices from a *Grin2D*-WT mouse aged P21 (gray trace) and *Grin2D*-null mouse aged P18 (black trace). Responses are scaled to the peak WT response. Scale bars are 500 pA and 20 s. **(C)** Bar graph shows the quantified desensitization in response to 10 mM NMDA (data points from individual cells are superimposed; ^∗^*P* = 0.026, Mann–Whitney test). Data in **(C)** are from seven *Grin2D*-WT mice and six *Grin2D*-null mice.

In order to investigate extrasynaptic NMDAR expression in *Grin2D*-null mice, we used three approaches. First, we applied NMDA at a range of concentrations to the bath perfusion solution, to obtain a concentration-response curve for NMDA-activated whole cell current (**Figure 5A**). The fit of the data gave a maximum current response (I_max_) and a concentration giving 50% of the maximum current response (EC_50_) in SNc-DA neurons from P21 mice. In WT mice, I_max_ was 2056 ± 155.9 pA and EC_50_ was 0.0837 ± 0.0438 mM. In *Grin2D*-null mice, I_max_ was 2913 ± 160.2 pA and EC_50_, 0.194 ± 0.0755 mM, significantly different to WT (both *P* = 0.0001, *F*-test). Desensitization of NMDARs involves a conformational change in the receptor resulting from agonist binding, which reduces current flow through the channel, and may constitute a negative feedback mechanism to prevent excitotoxicity (Zorumski et al., 1990). We observed that high concentrations of steady state NMDA (10 mM) caused significantly more desensitization in SNc-DA neurons from *Grin2D*-null mice (72.4 ± 3.9%, *n* = 7) compared with WT mice (55.1 ± 3.2%, *n* = 7; *P* = 0.026, Mann–Whitney test; **Figures 5B,C**).

**FIGURE 6:**
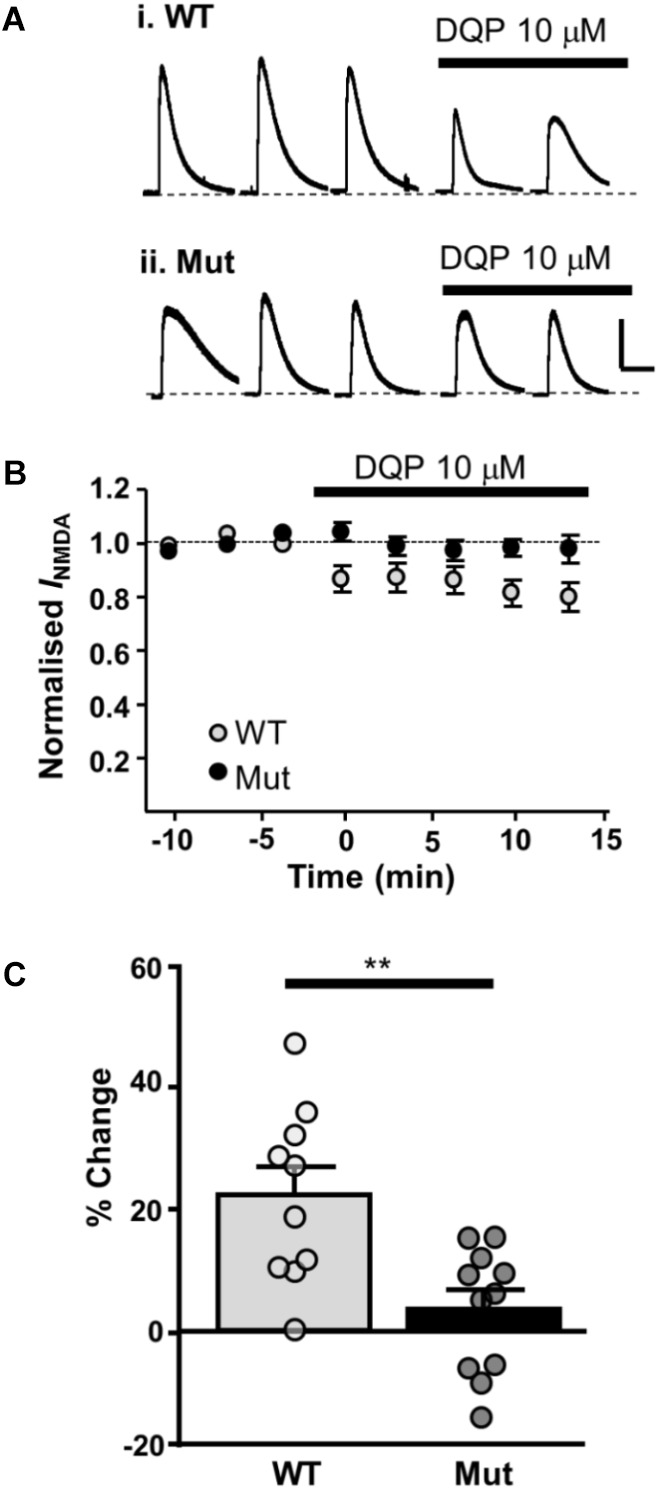
Whole cell NMDAR currents show reduced sensitivity to DQP-1105 in *Grin2D*-null mice. **(A)** Example recording from SNc-DA neurons in slices from (i) *Grin2D* WT mouse aged P19 and (ii) *Grin2D*-null (Mut) mouse aged P17 before and during perfusion with DQP-1105 (10 mM). Scale bar applies to both recordings. **(B)** Time course of the effect of DQP-1105 in WT mice (*n* = 10) and *Grin2D* null mice (*n* = 11). **(C)** Bar graph shows the % inhibition with DQP in WT versus *Grin2D*-null mice (data points from individual cells are superimposed; ^∗∗^*P* = 0.001, unpaired *t*-test). Data are from three *Grin2D*-WT mice, seven C57 mice and nine *Grin2D*-null mice.

Next, we investigated the effect of DQP-1105 on NMDA-activated currents. NMDA (plus glycine, picrotoxin and tetrodotoxin) was applied for three control responses and then in the presence of DQP-1105 (10 μM). In slices from WT mice, DQP-1105 caused 21.9 ± 4.6% inhibition (*n* = 10); significantly less inhibition was seen in slices from *Grin2D*-null mice (3.2 ± 3.1%, *n* = 11; *P* = 0.001, unpaired one-tailed *t*-test; **Figure 6**).

**FIGURE 7:**
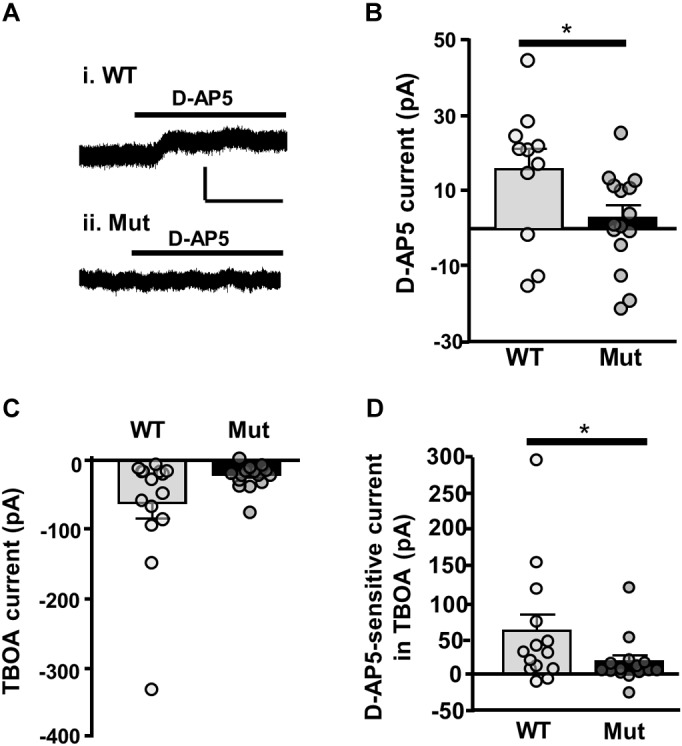
Tonic NMDAR current in *Grin2D*-null mice. **(A)** Example recording from SNc-DA neurons in slices from (i) a C57 WT mouse (P20) and (ii) a *Grin2D*-null (Mut) mouse (P19). Scale bar (50 pA, 100 s) applies to both recordings. **(B)** Bar graph shows D-AP5-sensitive current recorded in WT and *Grin2D*-null mice (data points from individual cells are superimposed; ^∗^*P* = 0.04, Mann–Whitney test). **(C)** Bar graph shows TBOA-evoked current recorded in WT and *Grin2D*-null mice (data points from individual cells are superimposed; *P* = 0.22, Mann–Whitney test). **(D)** Bar graph shows D-AP5-sensitive component of TBOA-evoked current recorded in WT and *Grin2D*-null mice (data points from individual cells are superimposed; ^∗^*P* = 0.045, Mann–Whitney test). Data are from nine *Grin2D*-WT, thirteen C57 and twenty five *Grin2D*-null mice.

Finally, we looked at tonic NMDAR current activated by ambient extracellular glutamate, which has been attributed to current mediated by extrasynaptic NMDARs (Le Meur et al., 2007). To determine whether GluN2D-containing NMDARs contribute to tonic NMDAR current, we measured the amplitude of D-AP5 (50 μM) sensitive baseline current in SNc-DA neurons from P21 WT and *Grin2D*-null mice. In SNc-DA neurons from *Grin2D*-null mice this was 2.8 ± 3.3 pA (*n* = 15), significantly less current than that seen in WT mice (15.6 ± 5.4 pA, *n* = 11; *P* = 0.043, Mann–Whitney test; **Figures 7A,B**). The glutamate transporter inhibitor TBOA (30 μM) was added to increase ambient levels of glutamate; TBOA was applied with LY 341495 (200 nM), an antagonist of Group II mGluRs (increased extracellular glutamate caused by the application of TBOA activates mGluRs and inhibits presynaptic glutamate release, leading to significant increases in inward current in SNc-DA neurons; Wild et al., 2015). Although TBOA-evoked current was smaller in slices from *Grin2D*-null mice (19.7 ± 4.8 pA, *n* = 15), it was not significantly different to that seen in WT mice (60.9 ± 22.1 pA, *n* = 15; *P* = 0.22, Mann–Whitney test; **Figure 7C**). However, the sensitivity of TBOA-evoked current to block by D-AP5 was reduced in *Grin2D*-null mice (18.3 ± 8.4 pA, *n* = 15) compared with WT mice (60.2 ± 21.9 pA, *n* = 14; *P* = 0.045, Mann–Whitney test; **Figure 7D**).

## Discussion

Overall, the data reported above indicate that *Grin2D*-null mice, with disrupted GluN2D expression, display an altered synaptic and extrasynaptic NMDAR subunit profile in SNc-DA neurons, supporting a functional role for GluN2D at glutamatergic synapses onto these cells, and a functional contribution in responsiveness to ambient or unusually high levels of extrasynaptic glutamate. *Grin2D*-null mice have a mild phenotype, they grow and mate normally and exhibit no major structural brain abnormalities (Ikeda et al., 1995; Miyamoto et al., 2002). In this study, we found the overall expression of NMDARs to be unaffected by the *Grin2D* mutation. None-the-less, as with all genetically modified models, there is a possibility of uncontrolled and unexplored effects.

### Altered Properties of Synaptic NMDARs in *Grin2D*-Null Mice

In order to evaluate whether overall synaptic NMDAR expression is altered in the genetically modified animals, AMPAR/NMDAR current ratios (A/N ratios) were measured, as the expression of AMPARs should not be altered in *Grin2D*-null mice. Both NMDAR and AMPAR numbers change during development (Wu et al., 1996; Petralia et al., 1999) but there was no obvious relationship between A/N and age. Genotype had no effect on the A/N ratio at P7 or P21, indicating that removal of the GluN2D subunit does not alter the amplitude of current passing through synaptic NMDARs. It is therefore likely that constitutive expression of synaptic NMDARs remains unaltered in *Grin2D*-null mice. Furthermore, there was no indication that the Mg^2+^-sensitive block of NMDAR-EPSCs at hyperpolarized membrane potentials was altered in SNc-DA neurons from *Grin2D*-null mice. These results suggest either that GluN2D is not present in synaptic NMDARs of WT mice, or that GluN2D is ordinarily present at a low level, such that it does not have a profound effect on the current-voltage relationship, and is replaced by another GluN2 subunit at synapses in *Grin2D*-null mice.

To further investigate the contribution of GluN2D subunits to NMDAR-EPSCs, subunit-preferring pharmacological inhibitors were applied to synaptic NMDAR-EPSCs. The small and variable effect (10–40%) of the GluN2D-preferring DQP-1105 on NMDAR-EPSCs in slices from WT mice is consistent with a population of GluN2D-containing NMDARs that might vary between dopamine neurons and is on average small (20–25%). In slices from *Grin2D*-null mice, DQP-1105 caused significantly less inhibition than in WT mice at both ages tested. However, it should be noted that even in Grin2D null mice there was a variable degree of inhibition; this could be due to changes in NMDAR-EPCS amplitude that occur independently of DQP over the time course of the recording, or due to a degree of non-selectivity of DQP in inhibiting NMDARs composed of other subunits, most likely GluN2B.

If GluN2D subunits are replaced with GluN2B subunits in *Grin2D*-null mice, then an increased inhibition with a GluN2B-preferring antagonist would be expected. Ifenprodil is an ideal compound to use in SNc-DA neurons in which the only GluN2 subunits present are likely to be GluN2B and GluN2D, as its affinity for GluN2D is low (IC_50_ of 76 μM for GluN2D, versus 0.1 μM for GluN2B; Hess et al., 1998). It should be noted, however, that ifenprodil does not cause complete inhibition of even a pure population of GluN2B-containing NMDARs; the maximum inhibition is ∼90% (Williams, 1993). In slices from both P7 and P21 *Grin2D*-null mice there was a significantly greater percentage inhibition of NMDAR-EPSCs by ifenprodil compared with slices from WT mice (summarized in **Table 1**), suggesting that in mice lacking the GluN2D subunit, GluN2D is replaced by GluN2B within the NMDAR. It seems likely that any triheteromeric GluN1/GluN2B/GluN2D NMDARs are replaced with diheteromeric GluN1/GluN2B receptors in *Grin2D*-null mice. The increase in ifenprodil inhibition in *Grin2D*-null mice compared to WT was approximately 16% at both age groups, and it is possible that this difference accounts for the proportion of GluN2D-mediated synaptic current in WT animals, taking into account the incomplete blocking action of ifenprodil.

The weighted decay time constant of control NMDA-EPSCs in WT mice at P7 (∼109 ms) and at P21 (∼75 ms) are comparable to those reported for rat NMDAR-EPSCs at P7 (∼150 ms) and P21 (∼70 ms) (Brothwell et al., 2008), and similarly showed no significant change in ifenprodil. It was suggested that the NMDAR-EPSC in rat SNc-DA neurons does not have a significantly altered decay time constant in control and ifenprodil because they are composed of similar subunits (Brothwell et al., 2008). If this is the case, one might expect a change in the decay time constant between WT and *Grin2D*-null mice; however, if synaptic NMDARs are triheteromers composed of GluN1/GluN2B/GluN2D subunits in WT mice, the GluN2B subunit may be the main determinant of the decay time constant, and removal of the GluN2D subunit may have little effect on the decay kinetics.

Overall, in mice lacking GluN2D, there was significantly less inhibition by DQP-1105 and significantly more inhibition by ifenprodil (**Table 1**): together this suggests that GluN2D is present at the synapse in WT mice, and that in *Grin2D*-null animals the GluN2D subunit is replaced by GluN2B within synaptic NMDARs; however, some caution is needed due to the small and variable effect of DQP-1105.

### High Concentrations of NMDA Elicit Larger Currents in *Grin2D*-Null Mice

The dose-response data obtained from bath application of NMDA appears to show no difference based on genotype at low or moderate NMDA concentrations, which suggests that numbers of NMDARs across the cell are similar. Interestingly however, toward their upper plateaus the curves separated: *Grin2D*-null mice displayed larger peak current in response to 1–10 mM NMDA than WT mice. If GluN2B-containing NMDARs are indeed replacing GluN2D-containing NMDARs, it is likely that due to the larger peak current and shorter timeframe required for GluN2B to become ready to reopen (at peak agonist concentrations, where immediate reopening is likely), its presence confers a capability for greater conductance at the highest levels of agonist saturation, despite a lower agonist affinity (Hess et al., 1998; Wyllie et al., 2013). GluN2D on the other hand has a lower conductance and slower channel kinetics (a deactivation time of around 2000 ms to, as opposed to around 200 ms for GluN2B), meaning that immediate reactivation is not possible. Therefore GluN2D, with its lower sensitivity to Mg^2+^ block and higher affinity for glutamate, is ideally suited to maintaining a baseline current flow in response to low levels of ambient glutamate, but its presence in a triheteromeric receptor may attenuate peak current flow in response to high agonist concentrations. Desensitization in response to a high NMDA concentration was also significantly increased in mice lacking GluN2D. Although these measurements were made during steady-state application of NMDA, such that some pre-desensitization of NMDARs may occur leading to underestimation, this is none-the-less consistent with the presence of GluN2D-containing NMDARs in slices from WT mice and their replacement with GluN2B-containing NMDARs in *Grin2D*-null mice, as GluN2B subunits desensitize to a greater extent than GluN2D (Wyllie et al., 1998; Vance et al., 2011; Wyllie et al., 2013).

Confirming the presence of GluN2D-containing NMDARs across the whole-cell profile, NMDA-activated currents were inhibited by DQP-1105 to a similar degree as NMDAR-EPSCs in slices from WT mice, and the effect of DQP-1105 was significantly reduced in slices from *Grin2D*-null mice. If GluN2D-containing NMDARs were enriched in either synaptic or extrasynaptic sites, an obvious difference in DQP-1105 inhibition of synaptic versus whole-cell NMDAR currents would have been expected.

### Tonic NMDAR-Mediated Currents Are Reduced in *Grin2D*-Null Mice

Extrasynaptic NMDARs can be activated by ambient extracellular glutamate and can maintain a tonic level of excitability (Sah et al., 1989; Angulo et al., 2004; Fellin et al., 2004). Desensitization of NMDARs is less than for AMPARs and therefore they are likely to mediate the majority of excitation in response to ambient glutamate (Iacobucci and Popescu, 2017). We have previously reported a small but significant D-AP5-sensitive tonic current that persists when neuronal firing is inhibited, indicating that NMDARs are activated by ambient extracellular glutamate which does not have an immediate action potential-dependent origin (Wild et al., 2015). Here, we show that in slices from mice lacking the GluN2D subunit there was significantly less D-AP5-sensitive tonic current in comparison to WT mice. This suggests that GluN2D-containing NMDARs have a role in mediating tonic NMDAR activity in response to ambient glutamate in SNc-DA neurons. The difference in D-AP5-sensitive current observed between genotypes may result from the increased glutamate affinity and low Mg^2+^ block characteristics conferred by the presence of GluN2D which allows it to contribute significantly to baseline levels of current in response to low levels of ambient glutamate; a function which has previously been predicted (Wyllie et al., 1998). This sensitivity of GluN2D to ambient glutamate may have a function in maintaining a tonic level of excitability in SNc-DA neurons, and/or in allowing modulation of neuronal excitability by glial cells.

Pharmacological inhibition of glutamate transporter activity using TBOA causes an increase in tonic NMDAR activity (Wild et al., 2015). Application of TBOA increased inward current in both WT and *Grin2D*-null mice, but significantly less TBOA-evoked current was sensitive to D-AP5 in slices from *Grin2D*-null mice, suggesting that GluN2D-containing NMDARs also mediate responses under conditions of glutamate transporter blockade. This is further evidence that GluN2D might be a good target for inhibition in neurodegenerative illnesses (such as PD) which may include glutamate transporter dysfunction as part of their pathophysiology (Lipton, 2004; Kotermanski and Johnson, 2009; Emre et al., 2010; Wild et al., 2013, 2015; Assous et al., 2014; Jensen et al., 2015).

## Conclusion

These results support the idea that GluN2D subunits are expressed within NMDARs both at synapses and across the surface of SNc-DA neurons. GluN2D-containing NMDARs appear to have a role in the detection of presynaptic glutamate release as well as ambient extracellular glutamate. Whilst they might mediate larger currents in response to reuptake dysfunction, their presence may also impose a lower maximum NMDAR-mediated current influx. GluN2D-containing NMDARs thus have diverse roles in SNc-DA neurons which may serve both to maintain normal function and protect the cell in potentially pathological conditions. The contribution of GluN2 subunit composition to essential NMDAR function is of considerable interest with regards to synaptic transmission and plasticity, and indeed neurodegenerative illness. The GluN2D subunit has been implicated as a potential target in preventing NMDAR-mediated excitotoxicity, particularly that which may contribute to ongoing degeneration in Parkinson’s Disease (Kotermanski and Johnson, 2009). In addition, subtle contributions of GluN2D to normal NMDAR function in SNc-DA neurons, as reported here, may also facilitate understanding of NMDAR function in other neurons which express GluN2D in mature animals, such as striatal and subthalamic neurons and hippocampal interneurons.

## Author Contributions

PM and SJ carried out the experiments, analyzed the data, and wrote the manuscript. MM provided the Grin2D mice.

## Conflict of Interest Statement

The authors declare that the research was conducted in the absence of any commercial or financial relationships that could be construed as a potential conflict of interest.
